# A Digital PCR Method Based on Highly Specific Taq for Detecting Gene Editing and Mutations

**DOI:** 10.3390/ijms241713405

**Published:** 2023-08-29

**Authors:** Bo Li, Junhao Liu, Qilai Huang

**Affiliations:** Shandong Provincial Key Laboratory of Animal Cell and Developmental Biology, School of Life Sciences, Shandong University, Qingdao 266237, China

**Keywords:** get-dPCR, highly specific Taq, gene editing efficiency, cancer gene detection

## Abstract

Digital PCR (dPCR) has great potential for assessing gene editing or gene mutation due to its ability to independently inspect each DNA template in parallel. However, current dPCR methods use a fluorescence-labeled probe to detect gene variation events, and their ability to distinguish variated sequences from the wild-type sequence is limited by the probe’s tolerance to mismatch. To address this, we have developed a novel dPCR method that uses a primer instead of a probe to sense gene variation. The enhanced Taq DNA polymerase in the PCR system has a high mismatch sensitivity, which enables our dPCR method to distinguish gene mutations from wild-type sequences. Compared to current dPCR methods, our method shows superior precision in assessing gene editing efficiency and single-base DNA mutation. This presents a promising opportunity to advance gene editing research and rare gene mutation detection.

## 1. Introduction

CRISPR/Cas9 genome editing technology has undergone rapid development and is currently being utilized in scientific research on animals [1,2,3,4], plants [5,6], and microorganisms [7,8]. When conducting a genome editing experiment, it is usually important to assess the effectiveness and specificity of single-guide RNAs (sgRNAs). Several techniques have been reported to determine the efficiency of genome editing [9], including denaturation-based techniques [10,11,12,13], Sanger DNA sequencing [14,15], next-generation sequencing [16,17,18,19,20,21], and direct PCR-based quantification [22,23,24,25,26,27,28]. However, techniques such as a surveyor nuclease assay that rely on denaturation can only provide a semi-quantification of the editing efficiency and suffer from high background signals. Sanger sequencing methods can become quite expensive when dealing with a large number of samples, while next-generation sequencing methods are quite time-consuming.

Digital PCR (dPCR) technology amplifies and detects individual template DNA molecules in massive droplets or microwells [29,30]. Benefitting from its power in absolute gene quantification, dPCR has been widely used in genetic analysis, clinical diagnosis and therapy applications [31,32,33,34,35]. In addition, the ability to inspect each DNA template independently makes it a powerful tool in gene editing or gene mutation assessment. The present dPCR method for gene-editing frequency detection, called gene-editing frequency digital PCR (GEF-dPCR), employs two probes within a single amplicon to detect nonhomologous end-joining (NHEJ)-affected alleles [23]. The “NHEJ-sensitive probe” can selectively match with the wild-type allele but not the NHEJ-mutated allele, while the “NHEJ-insensitive probe” can match both. Ideally, the proportion of wild-type and indel sequence can be quantified based on the double-positive and single-positive events. However, the ability of the NHEJ-sensitive probe to distinguish NHEJ-mutated alleles from wild-type alleles is not completely accurate. The NHEJ-mutated alleles can still produce false-positive fluorescent signals from the NHEJ-sensitive probe, and lead to heavy “raindrops” between positive and negative points [23], making it difficult to accurately define positive events and thus affecting the accuracy of the indel frequency analysis. Even though the application of a locked nucleic acid (LNA) probe can enhance the detection accuracy, “raindrops” still present heavily with indel templates in dPCR analysis [36].

To address this issue, we have developed a new digital PCR method, named “get-dPCR” (genome editing test dPCR) to assess gene editing efficiency and gene variation. This method utilizes a specific PCR primer, known as “watching primer” [24], which is designed to cover the NHEJ site and allow its 3′ end to span over 3–5 bases across the cutting site. Therefore, the watching primer becomes indel-sensitive and can distinguish mutated NHEJ alleles from the wild-type allele. The success of this technique is largely defined by the sensitivity of Taq DNA polymerase to primer/template mismatches at the 3′ end [37]. Hence, we use an enhanced Taq polymerase generated previously through molecular evolution to improve the ability to discriminate indels from wild sequences [38]. This enhanced Taq DNA polymerase has three amino acid substitutions, S577A, W645R, and I707V, that endow this variant with improved sensitivity to indel–derived primer/template mismatches. The get-dPCR technique can accurately measure the frequency of indels, even when it is as low as 1%. It shows a high accuracy and sensitivity, making it an ideal method. Additionally, this method exhibits excellent performance in single-base gene mutation detection. By using a primer with the mutant nucleotide located at the 3′ end, it is possible to distinguish between wild-type and mutant DNA with complete accuracy. It has great application value and broad application prospects in the fields of genome editing and genetic analysis, including the genotyping analysis of mutation sites and the detection of circulating tumor DNA.

## 2. Results

### 2.1. Ability of GEF-dPCR to Assess Indels

**Figure 1:**
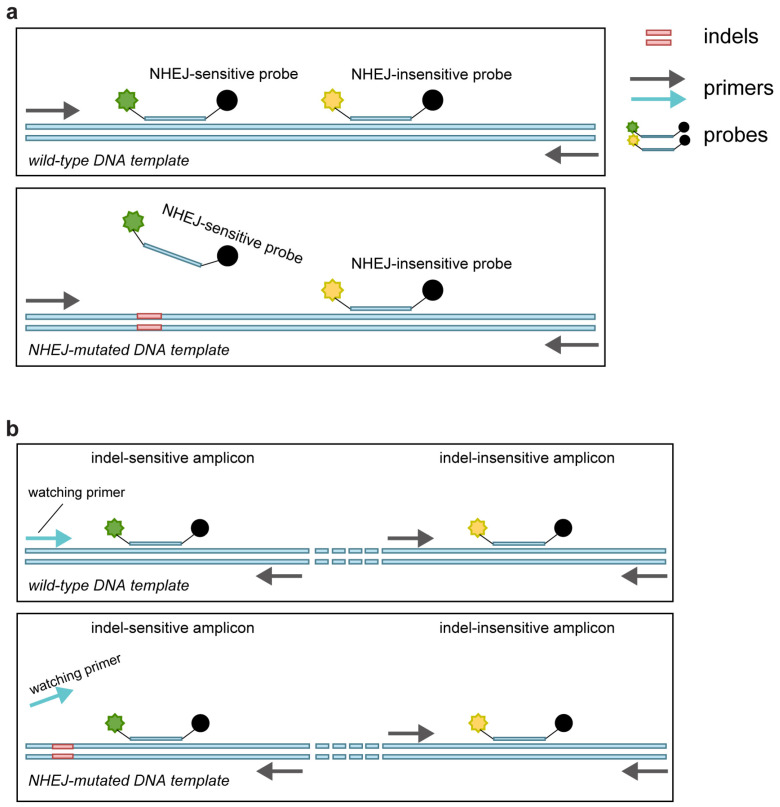
Schematic diagram of the principle of the GEF-dPCR assay and get-dPCR assay. (**a**) GEF-dPCR assay contains one NHEJ-sensitive probe and one NHEJ-insensitive probe in a single amplicon. The wild-type target sequence generates both fluorescence signals, while the indel sequence only generates fluorescence of the NHEJ-insensitive probe. (**b**) The get-dPCR assay has one indel-sensitive amplicon and one adjacent indel-insensitive amplicon. The watching primer matches the wild-type target sequence, with the cut site located at the 3′ end.

We utilized 26 plasmids containing different indel sequences on the *HOXB13* coding sequence (Supplementary Figure S1a) to mimic gene editing indels, as previously described [24]. The plasmid mixtures mimicking indel frequencies of 100%, 50%, 10%, 1%, and 0% were used to evaluate different gene-editing frequency assessment methods. We designed a HEX-labeled probe to sense the indels according to the GEF-dPCR assay protocol (Figure 1a) [23] and a watching primer that could discriminate indel sequences from the wild-type sequence, as previously described in the getPCR method [24].

**Figure 2:**
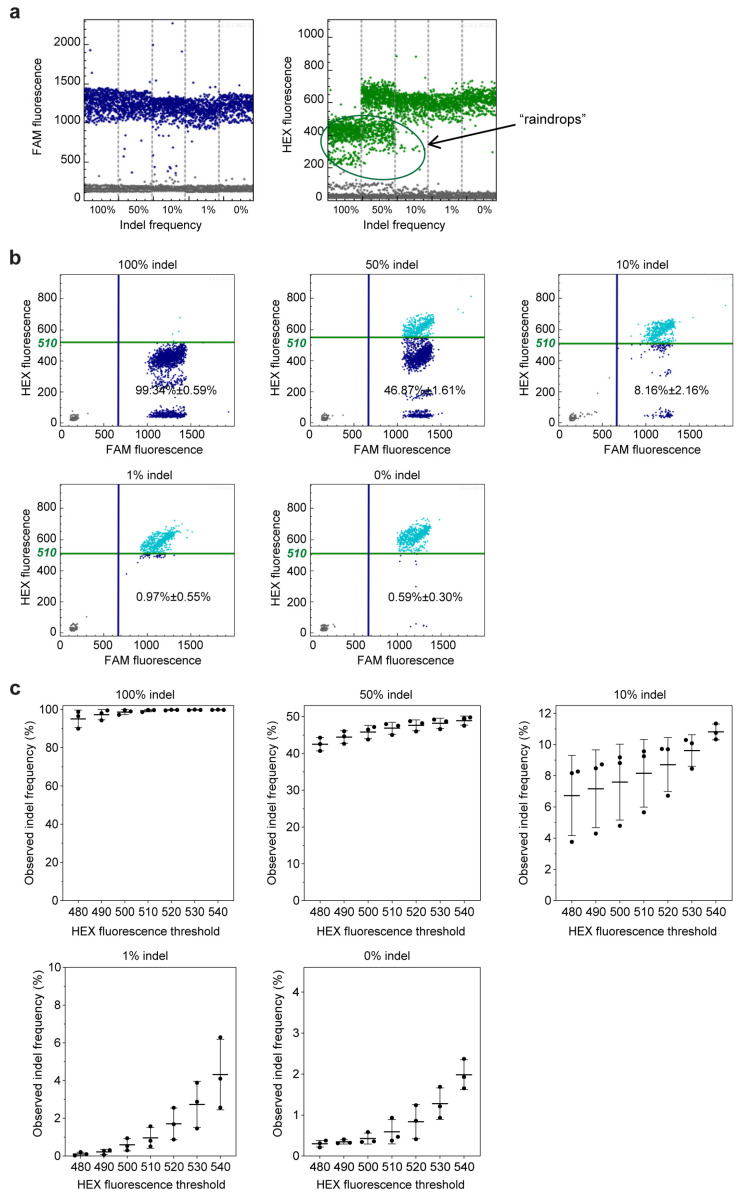
Editing frequency detection using GEF-dPCR assay. (**a**) One-dimensional diagram of the droplet fluorescence generated in the GEF-dPCR analysis of plasmid mixtures with an indel frequency of 100%, 50%, 10%, 1%, and 0%. Positive events of FAM signal, shown as blue dots, represent droplets producing the fluorescence of the NHEJ-insensitive probe, and positive events of the HEX signal (green dots in graphs) represent droplets producing the fluorescence of the NHEJ-sensitive probe. Negative events are plotted in gray color. (**b**) The two-dimensional diagram of the droplet fluorescence generated in the above GEF-dPCR analysis. Positive events are determined using a threshold of HEX fluorescence of 510. Double-positive events, shown as light blue dots, represent droplets indicating the wild-type sequence. Single-positive events of the FAM signal, shown as dark blue dots, represent droplets containing the NHEJ-mutated sequences. (**c**) The change in observed indel frequency along with shifting the threshold of HEX fluorescence.

Firstly, we conducted a test to evaluate the effectiveness of current digital PCR technology in assessing indels. The results showed that the positive droplets of the FAM signal, which indicate indel-insensitive amplification, could be easily distinguished (Figure 2a). However, the HEX signal, which represents indel-sensitive amplification, was also observed from the indel sequences, with a relevant lower level than the wild sequence (Figure 2a). These droplets of indel templates formed heavy “raindrops” between negative and positive events on the scatter plot. This indicates that the indel-sensitive probe has a poor ability to discriminate indel sequences from wild-type sequences.

To define the droplets of indel templates, a threshold has to be set between the positive wild-type droplet group and the raindrops group, as reported previously [23]. When the HEX fluorescence threshold was set as 510, the observed indel frequencies were 99.34% ± 0.59%, 46.87% ± 1.61%, 8.16% ± 2.16%, 0.97% ± 0.55%, and 0.59% ± 0.30% for the templates with 100%, 50%, 10%, 1%, and 0% indels, respectively (Figure 2b). To some extent, the observed indel frequencies may accurately represent the actual value. However, in practical use, there is usually no such control sample with a 100% indel frequency to aid in determining the threshold value. This makes threshold setting a challenging task, and the inherent subjectivity in the process may result in inaccurate and inconsistent results. Upon changing the threshold value, we observed obviously shifted indel frequency values for all five samples with 100%, 50%, 10%, 1%, and 0% indels (Figure 2c). This indicates that the GEF-dPCR analysis result is highly vulnerable to the threshold value due to the incomplete separation between these two groups of droplets.

### 2.2. get-dPCR Distinguishes Indels Clearly

**Figure 3:**
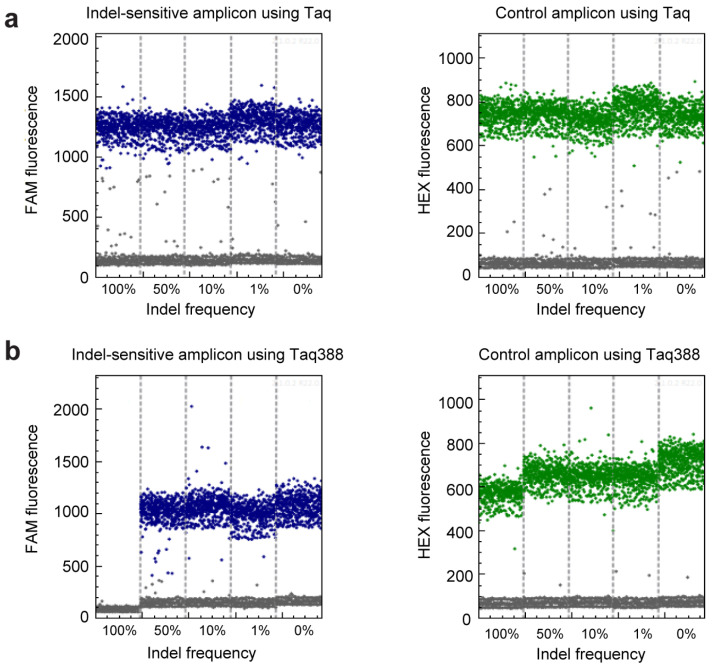
The ability of get-dPCR to distinguish indels. (**a**) One-dimensional diagram of the droplet fluorescence in the get-dPCR assay. Wild-type Taq DNA polymerase was used in the assay, with the plasmid mixtures with an indel frequency of 100%, 50%, 10%, 1%, and 0% as templates. (**b**) One-dimensional diagram of the droplet fluorescence in the get-dPCR analysis of the above plasmid mixtures using Taq388 DNA polymerase. The blue dots represent positive events of the FAM signal from the indel-sensitive probe, and the green dots represent positive events of HEX signal indicating the indel-insensitive replicon.

We then evaluated the get-dPCR method in assessing the indel frequency, using the primers and probes demonstrated in Figure 1b. Here, the FAM fluorescence signals the indel-sensitive amplicon, while the HEX fluorescence displays the indel-insensitive control amplicon. The watching primer used in the indel-sensitive amplicon had three 3′ end bases spanning the indel site to distinguish indel alleles from wild-type alleles effectively [24] (Supplementary Figure S1b). The plasmid mixtures mimicking indel frequency of 100%, 50%, 10%, 1%, and 0% were subjected to the get-dPCR assay, using wild-type Taq DNA polymerase (Figure 3a) or Taq388 [38], a highly specific Taq DNA Polymerase (Figure 3b). The results showed that when using the wild-type Taq for get-dPCR, both the wild target sequence and the indel sequences produced almost the same level of FAM fluorescence (Figure 3a). On the other hand, when using Taq388 for the get-dPCR assay, only the wild target sequence displayed FAM fluorescence, with no nonspecific FAM fluorescence from the indel sequences. This indicates that the get-dPCR assay using a highly specific Taq polymerase can completely distinguish indel sequences from the wild-type target sequence without producing raindrops.

### 2.3. get-dPCR Using Taq388 Quantify Indel Frequency Accurately

**Figure 4:**
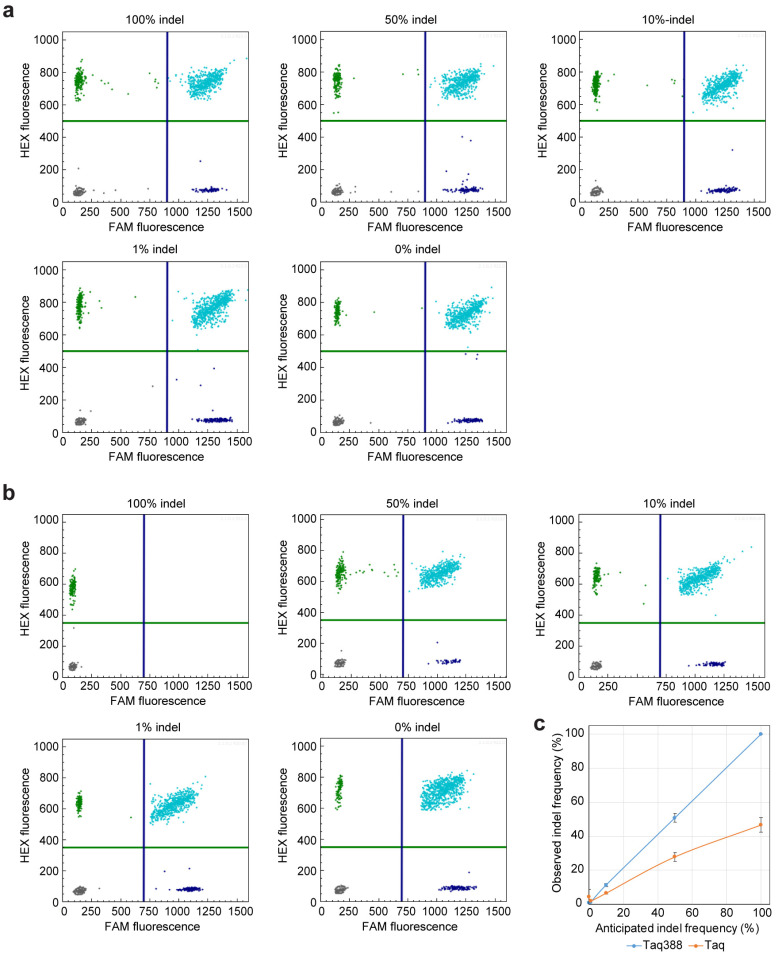
Determining editing frequency using the get-dPCR assay. (**a**) Two-dimensional diagram of the droplet fluorescence in the get-dPCR assay using wild-type Taq DNA polymerase. The plasmid mixtures with an indel frequency of 100%, 50%, 10%, 1%, and 0% were used in the assay. The FAM fluorescence represents the indel-sensitive amplicon, while the HEX fluorescence indicates the indel-insensitive replicon. (**b**) Two-dimensional diagram of the droplet fluorescence in the get-dPCR assay using Taq388. (**c**) Scatter plot showing the consistency between anticipated and observed indel ratios.

We further investigated the accuracy of the get-dPCR method in determining indel frequency. When using wild-type Taq DNA polymerase for the get-dPCR assay, the observed indel frequencies deviated greatly from the expected indel frequencies, with nearly half of the indel sequences misidentified as wild target sequence (Figure 4a). Specifically, the 100%, 50%, 10%, 1%, and 0% indel templates were determined to be 46.6%, 27.8%, 6.4%, 1.8%, and 4.4%, respectively. Notably, when using Taq388 for the get-dPCR assay, the observed indel frequencies for these plasmid mixtures were 100% ± 0%, 50.78% ± 2.55%, 11.2% ± 0.86%, 1.04% ± 0.19%, and 0.91% ± 0.36%, respectively (Figure 4b). This indicates that the get-dPCR method, armed with the highly specific Taq polymerase, can measure indel frequency accurately.

### 2.4. Gene Mutation Detection Using get-dPCR

Detecting gene mutations is critical for assessing disease risk, diagnosing and treating illnesses, and monitoring potential recurrences. Digital PCR technology has a great potential for detecting gene mutations, but its effectiveness is hindered by the challenge of distinguishing single-base mutations. In view of the above Taq388-based get-dPCR method showing a solid ability to distinguish indel gene variation, we next evaluated its performance in detecting single-base mutation.

For this analysis, we chose three gene mutation sites that are commonly used in cancer gene mutation detection [39,40]. These mutation sites are the *KRAS* c.35G > T (G12V), the *BRAF* c.1391G > T (G464V), and the *BRAF* c.1799T > A (V600E). According to targeted sequencing information from The Cancer Cell Line Encyclopedia (CCLE) [41], they are carried homozygous in the SW620 cell line, heterozygous in the MDA-MB-231 cell line, and heterozygous in the HT-29 cell line, respectively (Supplementary Table S2). The equal ratio mixture of genomic DNA from three cancer cells was subjected to a dPCR analysis, with the genomic DNA of the Lenti-X 293T cell line used as the wild-type sequence. The primers that distinguished a single-nucleotide mutation annealed to the mutation nucleotide with the 3′ end.

**Figure 5:**
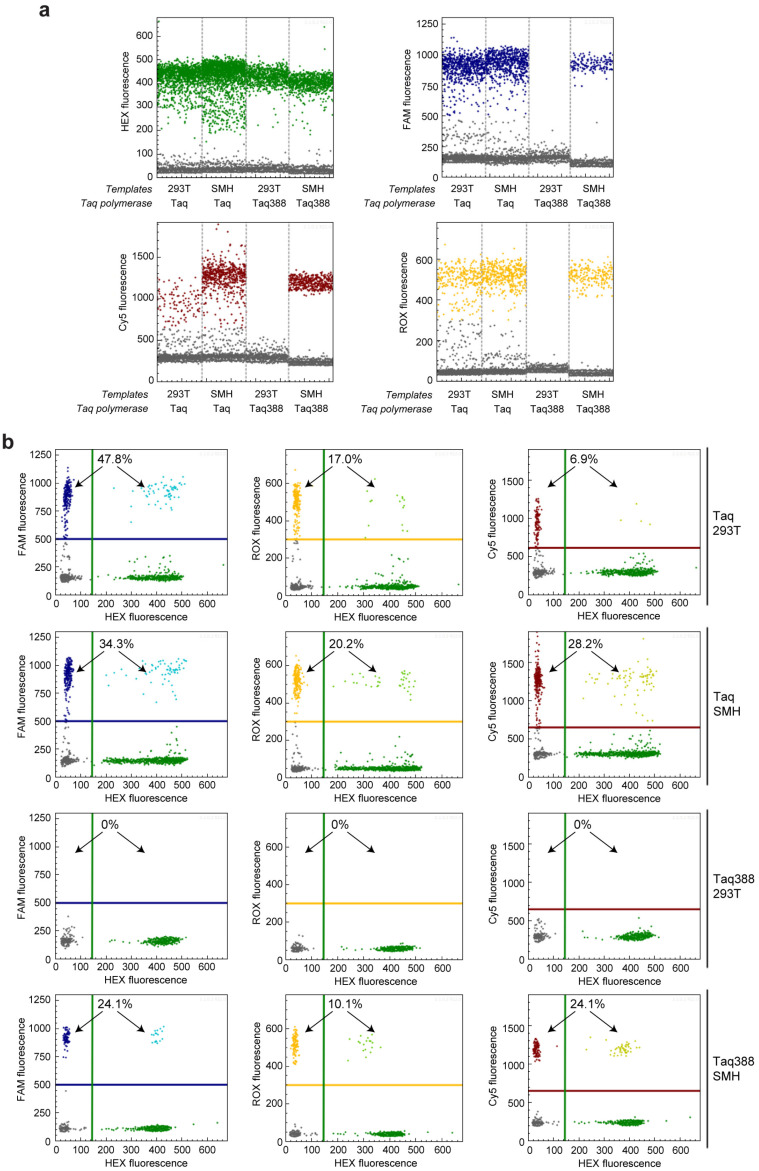
Detecting gene mutations using get-dPCR. (**a**) One-dimensional diagram of the droplet fluorescence generated in the get-dPCR analysis of three cancer gene mutations, *KRAS* c.35G > T, *BRAF* c.1391G > T, and *BRAF* c.1799T > A. The SMH template is the equal mixture of genomic DNA from SW620, MDA-MB-231, and HT-29 cells, which contain the three mutations, respectively. Genomic DNA from lenti-X 293T cells is negative for these three mutations. The green dots represent HEX fluorescence positive events, indicating the control amplicon targeting the *KRAS* 5′UTR region. The blue, red and orange dots represent positive events for the mutation-specific amplicon of *BRAF* c.1799T > A, *KRAS* c.35G > T, and *BRAF* c.1391G > T, respectively, indicated by the FAM, Cy5, and ROX fluorescence. (**b**) Two-dimensional diagrams of the droplet fluorescence in the above get-dPCR assay. The positive droplet ratios for FAM, Cy5, and ROX fluorescence are shown. The lines in blue, green, red, and orange represent the threshold for positive events of FAM, HEX, Cy5, and ROX fluorescence, respectively.

The dPCR reaction system consists of four amplicons in one single reaction, including one control amplicon in the *KRAS* 5′UTR region indicated with a HEX fluorescence, and three mutation-specific amplicons for detecting *KRAS* c.35G > T, *BRAF* c.1391G > T, and *BRAF* c.1799T > A, indicated using Cy5, ROX, and FAM fluorescences, respectively. When using wild-type Taq polymerase for the get-dPCR analysis, the wild-type target sequence of Lenti-X 293T cells also produced a Cy5, FAM, and ROX fluorescence which is intended to be specific to the three gene mutations (Figure 5). Notably, FAM and ROX fluorescences corresponding to *BRAF* c.1799T > A and *BRAF* c.1391G > T were almost as strong as that from the mutation-positive sequences (Figure 5a). Accordingly, the wild-type sequence of lenti-X 293T cells was misjudged to contain 6.9% *KRAS* c.35G > T, 17.0% *BRAF* c.1391G > T, and 47.8% *BRAF* c.1799T > A (Figure 5a). It indicates that get-dPCR using the wild-type Taq polymerase cannot distinguish single-base mutations. Notably, when using Taq388 for the get-dPCR assay, the lenti-X 293T genomic DNA did not generate any Cy5, FAM, or ROX fluorescence (Figure 5a). Accordingly, the HMH genomic DNA mixture was determined to carry 24.1% *KRAS* c.35G > T, 10.1% *BRAF* c.1391G > T, and 24.1% *BRAF* c.1799T > A. The values are consistent with the theoretical value calculated from the gene mutation genotypes of the three cancer cell lines. It strongly indicates that the Taq388-based get-dPCR technique has the capability to accurately distinguish single-base gene mutations.

## 3. Discussion

The broad adoption of genome editing using CRISPR/Cas9 has brought a huge impact to the field of life sciences. Accordingly, various techniques for detecting genome editing efficiency have been reported [24]. However, these methods are either complicated to use, costly in time and money, or insufficient in detection accuracy, especially in the case of low editing efficiency. In this study, by combining the power of dPCR technology, which inspects each template individually, and the high specificity of Taq388, we developed a digital PCR-based method named get-dPCR for detecting genome editing. This method can distinguish indel mutation completely from the wild-type target sequence and measure the indel frequency accurately. Moreover, the get-dPCR technique demonstrated the ability to distinguish single-base mutations with extreme clarity, as exemplified in the detection of three cancer mutations, *KRAS* c.35G > T, *BRAF* c.1391G > T, and *BRAF* c.1799T > A.

Compared with the present GEF-PCR, the get-dPCR technique has several advantages. Firstly, the highly specific Taq388 allows the mutation-specific primer to completely distinguish indel mutations to an extent superior to the indel-sensitive probes used in the GEF-PCR method. It enables the get-dPCR technique to accurately detect small indel mutations, even single-base substitutions produced using base editors. Secondly, the superior ability of get-dPCR to discriminate gene mutations effectively eliminates the occurrence of nonspecific signals known as “raindrops”. The raindrops can unavoidably occur in the GEF-PCR method and complicate the judgement of indel droplets. In addition, the superior capability of get-dPCR to distinguish gene mutations makes it a valuable tool for detecting cancer mutations.

On the other hand, there are several aspects that need attention when using get-dPCR. For example, designing effective primers and probes may still be a challenge for target sequences that are highly repetitive or rich in GC/AT. Even though primers can be designed routinely as described previously [24], it is recommended to conduct a gradient PCR to determine the optimal annealing temperature in order to obtain the best distinguishing capability. Moreover, the application of get-dPCR relies on the availability of expensive digital PCR instruments, which restricts the use of this technique to some degree.

In summary, the get-dPCR technique can accurately detect gene mutations, including indels and single-base gene mutations. It holds great potentials in measuring genome editing efficiency and in the genotyping of genome-editing offspring. Additionally, it is a valuable tool for detecting clinical cancer gene mutations and inspecting circulating tumor DNA.

## 4. Materials and Methods

### 4.1. Cell Culture

The SW620 cells (ATCC, Manassas, VA, USA, Cat#CCL-227), MDA-MB-231 cells (ATCC, Cat#HTB-26), HT-29 cells (ATCC, Cat#HTB-38), and Lenti-X 293T cells (Clontech Laboratories, TaKaRa Bio Company, Mountain View, CA, USA, Cat#632180) were cultured using Dulbecco’s Modified Eagle’s Medium (Gibco, New York, NY, USA, Cat#C11995500BT) supplied with 10% (*v*/*v*) FBS (Gibco, Cat#10270-106) and 1% penicillin-streptomycin (HyClone, Logan, UT, USA, Cat#SV30010) according to the manufacturer’s instruction. All the cells were maintained at 37 °C with 5% CO_2_ and were regularly checked for mycoplasma using a MycoBlue^TM^ mycoplasma detector kit according to the product manual (Vazyme, Nanjing, China, Cat#D101-01).

### 4.2. Plasmids

The plasmids containing wild-type *HOXB13* gene coding sequence and 26 indel-mutation variants were cloned previously in the pcDNA3.1 vector [24,42]. We mixed 26 plasmids with indel variants in equal amounts and considered them template DNA for the 100% indel frequency. This plasmid mixture was then combined with the wild-type construct to produce indel frequencies of 50%, 10%, and 1%. The wild-type and indel variant sequences are shown in Supplementary Figure S1a.

### 4.3. Genomic DNA

The genomic DNA was prepared from Lenti-X 293T, SW620, MDA-MB-231, and HT-29 cells using the TIANamp Genomic DNA Kit (TIANGEN, Beijing, China, Cat#DP304-03) according to the manufacturer’s instruction, and their cancer mutation information was retrieved from the BioPortal database “http://www.cbioportal.org/” (accessed on 14 November 2022) [43,44]. The genomic DNA of SW620, MDA-MB-231, and HT-29 cells were mixed equally to create an SMH genomic DNA mixture. Prior to the digital PCR analysis, the genomic DNA samples were sheared into fragments through 20 s of pulse-on sonication on a Bioruptor (Bioruptor Pico, Seraing (Ougrée), Belgium) at a DNA concentration of 10 ng/μL in 300 μL of TE buffer in a 1.5 mL Bioruptor^®^ Microtubes.

### 4.4. Primers and Probes

For the GEF-dPCR analysis, we used primers and probes that were designed according to a previous report [23]. A specific pair of primers were designed to amplify a 113 bp region that covers the NHEJ site. The HEX-labeled indel-sensitive probe was designed to span three bases across the NHEJ site with its 5′ end, while the indel-insensitive probe annealed to the adjacent region on the same amplicon. For analyzing the indel frequency using get-dPCR, two sets of primers and probes were designed for the indel-sensitive amplicon and the indel-insensitive amplicon, respectively, according to our previous getPCR method [24]. The 105 bp indel-sensitive amplicon used a FAM-labeled probe to produce a signal and a watching primer to discriminate indel modifications from wild-type sequences. The watching primer spanned 3–5 bases across the Cas9 nuclease cutting site at its 3′ end [24]. On the other hand, the indel-insensitive amplicon was located in the neighboring region unaffected by indels and used a HEX-labeled probe to generate a fluorescence signal. For the analysis of single-base DNA mutations, we designed watching primers for three gene mutations, *KRAS* c.35G > T, *BRAF* c.1799T > A, and *BRAF* c.1391G > T, respectively. The corresponding mutation was located at the 3′ end of each watching primer. The primer Tm value was calculated using the online Oligo Calc tool using the ”Salt Adjusted” parameter [45]. All the primer sequences are shown in Supplementary Table S1.

### 4.5. Conditions for dPCR

A typical 30 μL dPCR reaction system included 6 μL of 5 × TaqMan probe buffer, 50 fg of plasmid DNA or 5ng genomic DNA, 6 pmol of each primer, 3 pmol of each probe, and 0.6 μL of Taq polymerase (Small Turtle Technology, Shanghai, China, Cat#SCEHY001005-2000). The PCR reaction systems were then subjected to a digital PCR analysis on a Small Turtle Technology BioDigital QING-digital PCR system. Briefly, the reaction system was loaded into the digital PCR chip using the Fully Automatic Sample Processing System loader S200, and then cycled on the Cycler S200 instrument (Small Turtle Technology, Shanghai, China) with the following program: a preincubation at 50 °C for 10 min, an initial denaturation at 95 °C for 10 min, then 45 cycles of 95 °C for 30 s, 65 °C for 30 s, followed with a final holding at 25 °C. After the PCR reaction, the fluorescence signal was read and analyzed using a Biochip Reader Imager T200 (Small Turtle Technology, Shanghai, China).

## Data Availability

The data could be available from the corresponding author upon reasonable request.

## Supplementary Materials

The following supporting information can be downloaded at https://www.mdpi.com/article/10.3390/ijms241713405/s1.
