# Supplementary material for: A Digital PCR Method Based on Highly Specific Taq for Detecting Gene Editing and Mutations
Source: Int J Mol Sci. 2023 Aug 29;24(17):13405. doi: 10.3390/ijms241713405 (PMC10488114; doi:10.3390/ijms241713405)
Supplement: Supplementary file 1 [file Supplementary Figure S1.pdf › Supplementary Figure S1.pdf]

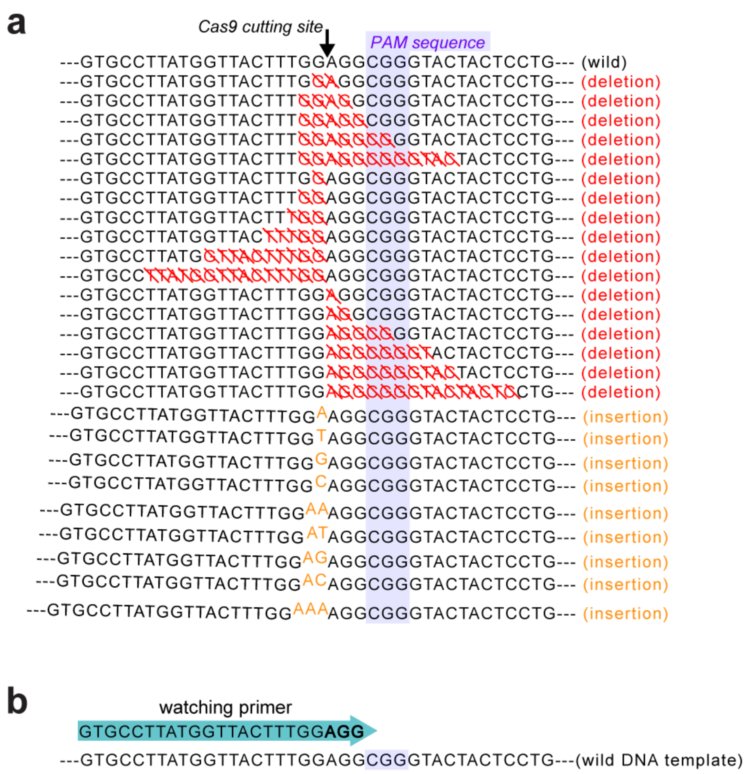

Supplementary Figure S1. Schematic illustration of indel sequence and primer design. (**a**) PAM sequence location and individual sequence of 26 indel gene variations on HOXB13 gene coding sequence. (**b**) the sequence and location of the indel-sensitive primer, containing three watching bases "AGG".
