# Supplementary material for: A Digital PCR Method Based on Highly Specific Taq for Detecting Gene Editing and Mutations
Source: Int J Mol Sci. 2023 Aug 29;24(17):13405. doi: 10.3390/ijms241713405 (PMC10488114; doi:10.3390/ijms241713405)
Supplement: Supplementary file 1 [file ijms-24-13405-s001.zip › Supplementary Tables.pdf]

Supplementary Table S1. Oligos for dPCR assay.

A. Oligos for GEF-dPCR analysis

	**sequence (5′ – 3′)**
Primer 1	CAGCTCCCGTGCCTTATGGTTAC
Primer 2	CTCCGCGGGGTACGCGGCCA
NHEJ-sensitive probe	HEX-TGGAGGCGGGTACTACTCCTGCCG-BHQ1
NHEJ-insensitive probe	FAM-ccctgtgcccaggcagccacc-BHQ1

B. Oligos for get-dPCR analysis in indel-frequency quantification

**indel-sensitive amplicon**	**sequence (5′ – 3′)**
Primer 1 (*watching primer*)	GTGCCTTATGGTTACTTTGGAGGC
Primer 2	CTCCGCGGGGTACGCGGCCA
Probe	FAM-ccctgtgcccaggcagccacc-BHQ1

**indel-insensitive amplicon**	**sequence (5′ – 3′)**
Primer 1	GTTCATCACCAAGGACAAGAGGCG
Primer 2	TGTTCTTCACCTTGGCGAGAACCT
Probe	HEX-CTCGGCAGCCACCAGCCTCTCG-BHQ1

C. Oligos for get-dPCR analysis in site-mutation detection

**KRAS c.35G>T mutation-sensitive amplicon**	**sequence (5′ – 3′)**
Primer 1 (*watching primer*)	AAGGCACTCTTGCCTACGCCAA
Primer 2	CACATTTTCATTATTTTTATTATAAGGCCTGCTG
Probe	CY5-AGCTCCAACTACCACAAGTT-MGB

**BRAF c.1799T > A mutation-sensitive amplicon**	**sequence (5′ – 3′)**
Primer 1 (*watching primer*)	AAATAGGTGATTTTGGTCTAGCTACAGA
Primer 2	CCATCCACAAAATGGATCCAGACAACTG
Probe	FAM-TCTCGATGGAGTGGGTCCC-MGB

**BRAF c.1391G>T mutation-sensitive amplicon**	**sequence (5′ – 3′)**
Primer 1 (*watching primer*)	TTGTAGACTGTTCCAAATGATCCAGATA
Primer 2	CGGGACTCGAGTGATGATTGGGAGA
Probe	ROX-TGTCCCACTGTAATCTGCCC-MGB

**mutation-insensitive amplicon**	**sequence (5′ – 3′)**
Primer 1	GCTCGGCCAGTACTCCCGGC
Primer 2	CCGCCGCCTTCAGTGCCTGC
Probe	HEX-CGCCATTTCGGACTGGGAG-3′ MGB

Supplementary Table S2. Mutation information of cancer cell lines

Cell line	Gene	Protein Change	Chromosome	HGVSc*	Mutation Type	Allele Frequency	Variant Reads	Ref Reads
SW620	KRAS	G12V	12	ENST00000311936.3:c.35G>T	Missense_Mutation	1	291	0
HT-29	BRAF	V600E	7	ENST00000288602.6:c.1799T>A	Missense_Mutation	0.244763	222	685
MDA-MB-231	BRAF	G464V	7	ENST00000288602.6:c.1391G>T	Missense_Mutation	0.586667	88	62

* Human Genome Variation Society (HGVSc)

Information obtained from the cBioPortal Database
